# The definition of low wall shear stress and its effect on plaque progression estimation in human coronary arteries

**DOI:** 10.1038/s41598-021-01232-3

**Published:** 2021-11-11

**Authors:** Eline M. J. Hartman, Giuseppe De Nisco, Frank J. H. Gijsen, Suze-Anne Korteland, Anton F. W. van der Steen, Joost Daemen, Jolanda J. Wentzel

**Affiliations:** 1 Department of Cardiology, Erasmus MC Rotterdam, P.O. Box 2040, 3000 CA Rotterdam, The Netherlands; 2 PoliToBIOMed Lab, Department of Mechanical and Aerospace Engineering, Politecnico di Torino, Turin, Italy; 3 Erasmus MC Rotterdam, Dr. Molewaterplein 40, 3031 GD Rotterdam, The Netherlands

**Keywords:** Biomedical engineering, Cardiology, Cardiovascular diseases, Vascular diseases, Atherosclerosis

## Abstract

Wall shear stress (WSS), the frictional force of the blood on the vessel wall, plays a crucial role in atherosclerotic plaque development. Low WSS has been associated with plaque growth, however previous research used different approaches to define low WSS to investigate its effect on plaque progression. In this study, we used four methodologies to allocate low, mid and high WSS in one dataset of human coronary arteries and investigated the predictive power of low WSS for plaque progression. Coronary reconstructions were based on multimodality imaging, using intravascular ultrasound and CT-imaging. Vessel-specific flow was measured using Doppler wire and computational fluid dynamics was performed to calculate WSS. The absolute WSS range varied greatly between the coronary arteries. On the population level, the established pattern of most plaque progression at low WSS was apparent in all methodologies defining the WSS categories. However, for the individual patient, when using measured flow to determine WSS, the absolute WSS values range so widely, that the use of absolute thresholds to determine low WSS was not appropriate to identify regions at high risk for plaque progression.

## Introduction

Wall shear stress (WSS), the frictional force of the blood flow exerting on the vessel wall, plays a vital role in the natural history of atherosclerosis. Fusion of multimodality imaging and computational fluid dynamics allowed comprehensive detailed research on WSS related plaque progression in coronary arteries. Using this methodology, multiple studies have shown the relationship between low WSS and atherosclerosis initiation and plaque progression in coronary arteries of humans and animal models of atherosclerosis^1–6^*.* These WSS patterns mostly explained the patched distribution of plaques throughout the arteries^7^*.* Therefore, identifying low, mid and high WSS in the coronary system contributes to understanding of the pathophysiology of plaque progression and plaque rupture.

Previous research used different methodologies to define WSS categories. Thereby different approaches were used to divide WSS into three groups: low, mid, and high WSS regions at the vessel wall^2,3,8^. To further explore the effect of WSS on plaque progression in clinical studies and to place the previous WSS research in context, it is crucial to understand the impact of the different approaches of dividing WSS into categories on this process.

One of the methodologies to determine low WSS regions is to use *vessel-specific* tertiles to acknowledge the observed heterogeneity in WSS and the patched distribution of plaques within  a single vessel. Thereby, one-third of the sectors within one vessel allocates as low WSS. When using this method, all vessels will have individual thresholds of low and high WSS^4,6,8^.

A second methodology often used to determine low, mid and high WSS thresholds is based on *study-specific* tertiles, allocating one-third of all the sectors of all vessels in the study as being exposed to low WSS and one-third exposed to mid and one-third to high WSS^2,9,10^. A large study used this study-specific thresholds methodology deducting low and high WSS thresholds based on their study. Later, these thresholds deducted from this previous study served as an absolute threshold in many studies that followed. Therefore, we classify this third methodology as *literature-based* thresholds^1,11–13^.

As WSS is determined by blood flow, vessel diameter, and curvature, and since we know that the three coronary arteries are located at different parts of the heart surface, this potentially can lead to differences in specific flow rates between the studied coronary artery type. Furthermore, some studies report on WSS and plaque progression in the LADs only^3^. Therefore, a fourth methodology was applied defining *vessel type-specific* thresholds, using tertiles per vessel type. For example, the one-third of sectors with the lowest or highest absolute WSS of all the LADs are used to determine low and high WSS for the LADs. Similarly, low and high WSS thresholds can be determined for the LCX and RCA.

It is still unclear how the definition of low, mid and high WSS impacts the predictive power of WSS for plaque progression. Therefore, in this current study, we used four different approaches to determine WSS thresholds within one existing dataset and investigated the WSS-related plaque progression and the predictive power of low WSS for plaque progression. The following four methods to determine WSS thresholds were compared: (A) subdivision of the WSS data into tertiles per vessel (*vessel-specific*), (B) subdivision of the WSS data into tertiles including all arteries with in the study (*study-specific*), (C) absolute thresholds based on the literature (*literature-based*) thresholds, (D) Three different thresholds per vessel type, i.e. dividing the sectors of each vessel type separately in tertiles (*vessel-type-specific*).

Furthermore, using these different methodologies to define WSS thresholds, in this study, the data were stratified according to three major coronary artery types to investigate the differences in the WSS-related plaque progression between the vessel types.

## Results

For 41 vessels (40 patients), a complete dataset with IVUS at baseline and follow-up, doppler data and the CT-scan was available. The patient characteristics are presented in Table 1. The mean age was 60 ± 9 years and 93% of the studied population was male. The median length of the ROI was 54 mm (39–62). After excluding regions with extensive calcification, a total of 10,205 45° × 1.5 mm sectors were used in the final plaque progression analysis. The median baseline plaque burden (PB) was 29% (16–46). In total, 15 LAD’s, 10 LCX’s and 16 RCA’s were present in the study. The characteristics of the three major vessels are presented in Table 2.

**Table 1:** Patient characteristics.

**N = 40 patients**	
*Clinical characteristics*	
Age, years	60 ± 8.6
Men, n (%)	37 (93%)
Body Mass Index (kg/m^2^)	27 ± 4.8
Diabetes Mellitus, n (%)	8 (20%)
Hypertension, n (%)	10 (25%)
Dyslipidemia, n (%)	18 (45%)
Current smoking, n (%)	10 (25%)
Positive family history, n (%)	13 (33%)
Previous MI, n (%)	8 (20%)
Previous PCI, n (%)	11 (28%)
LDL (mmol/L)	2.6 [2.1–3.2]
**Imaged study vessel N = 41 vessels**	
LAD, n (%)	15 (37%)
LCX, n (%)	10 (24%)
RCA, n (%)	16 (39%)

*MI* myocardial infarction, *PCI* percutaneous coronary intervention, *LDL* low dense lipid protein, *LAD* left anterior descending, *LCX* left coronary circumflex, *RCA* right coronary artery.

**Table 2:** Vessel characteristics.

	LAD	LCX	RCA	*p*
Length ROI	53 mm [48–62]	38 mm [25–60]	55 mm [40–58]	NS
Baseline PB	27% [16–42]	29% [17–46]	30% [15–47]	* < 0.05
Absolute WSS ROI	0.56 Pa [0.35–1.07]	0.87 Pa [0.56–1.46]	0.92 Pa [0.57–1.60]	< 0.05
% sectors (no calcification) < 1 Pa	72%	59%	54%	< 0.05
% sectors showing progression (> 90th percentile delta PB)	8%	15%	11%	< 0.05

*ROI* region of Interest, *PB* plaque burden, *WSS* wall shear stress, *LAD* left anterior descending, *LCX* left coronary circumflex, *RCA* right coronary artery.

*Only significant for LAD versus LCX and LAD versus RCA.

**Figure 1:**
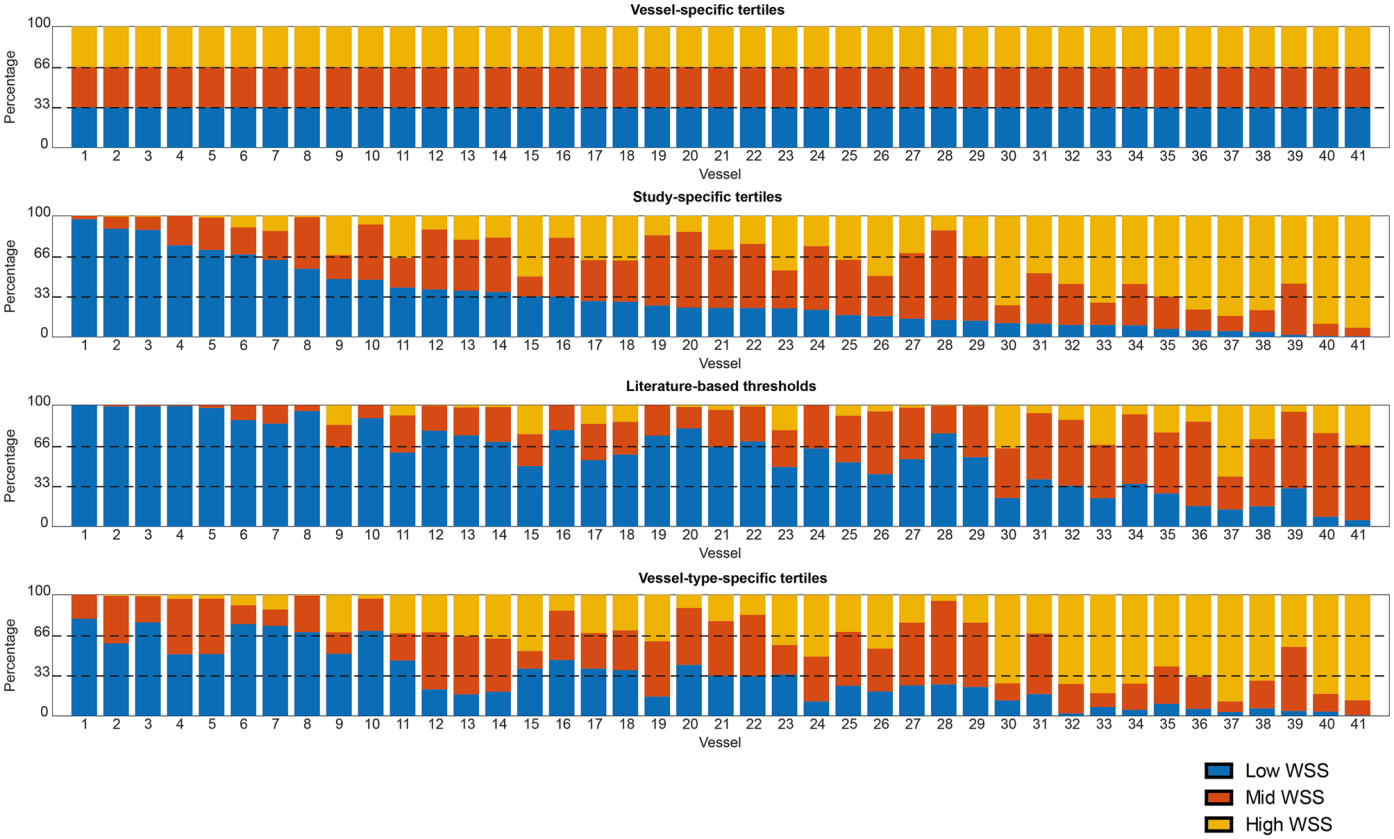
Overview of all individual study vessels (1–41) showing the WSS distribution for each vessel using the four different methodologies of WSS allocation. Sorted from left to right based percentage of low WSS in study-specific thresholds. Low WSS = blue, Mid WSS = orange, High WSS = yellow.

The WSS in the ROI was 0.77 Pa (0.46–1.37). In Fig. 1, for each individual vessel and each methodology to identify the WSS categories, the distribution of sectors exposed to low, mid and high WSS is presented. For the study-specific methodology, the absolute threshold for low WSS was < 0.60 Pa and high WSS > 1.14 Pa. Using this method, nearly half of the LAD sectors (48%) was identified as low WSS, whereas that percentage was much lower for the LCX and RCA, being 28% and 23.5% respectively. Using the thresholds based on literature (low < 1 Pa, high > 2.5 Pa), 62% of the sectors were characterized as low WSS; having 73% of the LAD, 56% of the LCX and 54% of the RCA being exposed to low WSS. All individual thresholds for the vessel-specific method are presented in the supplement Table. The average thresholds per vessel type for low and high WSS was for the LAD 0.43 Pa and 0.86 Pa, for the LCX 0.67 Pa and 1.27 Pa and RCA 0.68 Pa and 1.30 Pa.

Interestingly, even though the LAD presented with significantly lower average absolute WSS (Table 2), a wide range in WSS per vessel was observed. Therefore, the thresholds to demarcate low and high WSS in the LAD using the vessel-specific method were overlapping with the thresholds of the LCX and RCA (see supplement). Plaque growth of more than 15% PB, i.e. the 90th percentile of plaque progression of all sectors, was observed in 8% of the LAD, 15% of the LCX, and 11% of the RCA.

### WSS-related plaque progression

**Figure 2:**
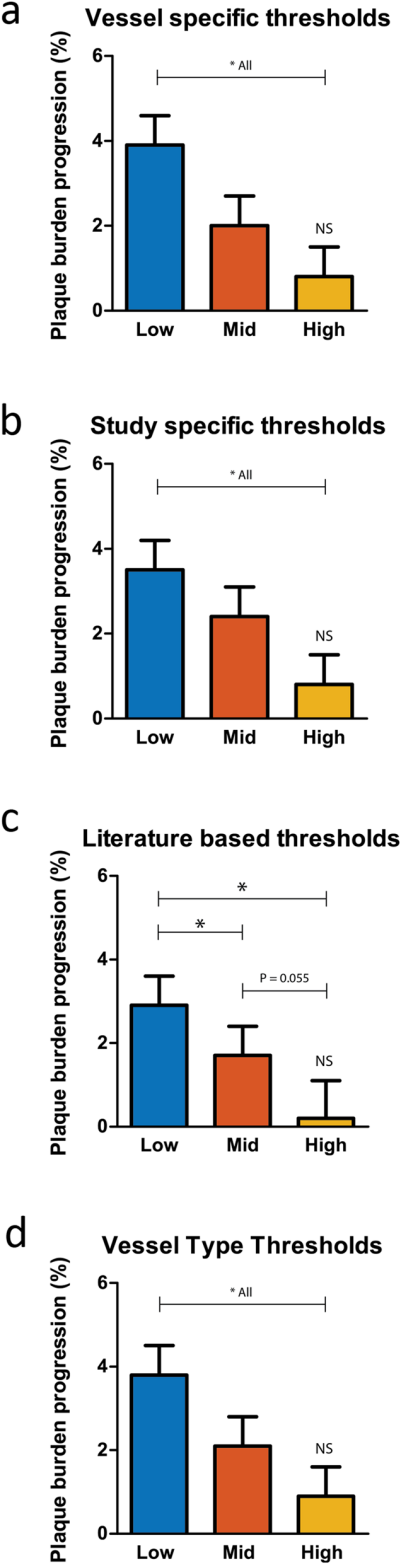
Wall shear stress distribution and plaque progression for the four different thresholding methodologies. *p < 0.05.

**Figure 3:**
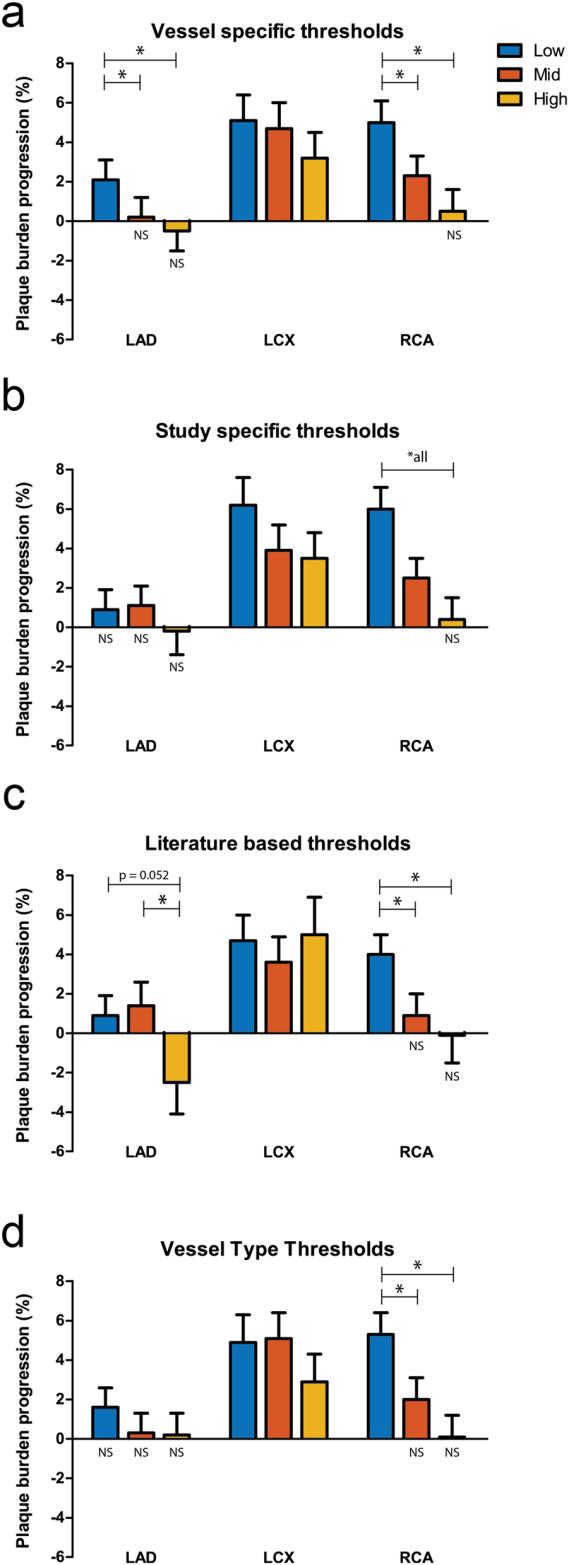
Wall shear stress distribution and plaque progression for the four different thresholding methodologies split up in the three coronary artery types. *p < 0.05.

The use of different WSS thresholds to define low, mid and high WSS had an effect on the predictive power of WSS for plaque progression. First, we report the estimated PB progression for sectors exposed to low, mid and high WSS. For vessel-specific WSS tertiles, the plaque progression of the sectors exposed to low WSS was significantly higher compared to the sectors exposed to mid and high WSS (Fig. 2a). (low: 3.9% 95%CI (2.5–5.2), mid: 2.0% 95%CI (0.7–3.3) high: 0.8% 95%CI (− 0.5–2.2), all *p* < 0.024. When analyzing the WSS related plaque growth of the different coronary vessels separately, this pattern was also visible in the LAD and RCA, showing significant difference between low WSS and mid/high WSS. The LCX did not show any differences in plaque progression between the three WSS groups (Fig. 3a).

When using all sectors within the study to define three equal groups of low, mid and high WSS (*study-specific*), also significant differences in plaque growth were found between sectors exposed to the low, mid and high WSS sectors (Fig. 2b). (low: 3.5% 95%CI (2.1–4.9), mid: 2.4% 95%CI (1.0–3.8) high: 0.8% 95%CI (− 0.6–2.3), all *p* < 0.05. Interestingly, after splitting the sectors based on vessel type, only the RCAs in our study demonstrated a similar pattern, which was fully disappeared for the LCX and LAD (Fig. 3b).

When defining the WSS categories using *literature-based* absolute thresholds, also significant differences were found between the three different WSS categories with the highest plaque growth for the low WSS sectors (Fig. 2c). ((low: 2.9% 95%CI (1.5–4.2), mid: 1.7% 95%CI (0.2–3.1) high: 0.2% 95%CI (− 1.6–2.1) *p* < 0.05. However, for the high WSS sectors no significant plaque growth was observed. For the different vessel types, hardly any plaque growth or differences in plaque growth for the sectors exposed to low, mid and high WSS could be demonstrated, except for the RCA (Fig. 3c).

Using the *vessel-type-specific* methodology to define the WSS categories, significant differences were found in plaque growth between the three different WSS categories (Fig. 2d) ((low: 3.8% 95%CI (2.4–5.1), mid: 2.1% 95%CI (0.8–3.5) high: 0.9% 95%CI (− 0.5–2.3)) *p* < 0.05. In a sub-analysis, the RCA showed significant differences in plaque growth between WSS categories, whereas no significant differences in plaque growth was observed in the LAD and LCX. (Fig. 3d).

Previous WSS studies did not correct for baseline plaque burden, however, today we recognize that regression to the mean, caused by the semi-automated analysis of the atherosclerotic plaque should be considered as a potential explanation for the findings of plaque changes over time. Hence, extra analyses correcting for baseline plaque burden are added in the supplement figures of this article (Supplement Figs. 2 and 3). Interestingly, only the vessel-specific method, with WSS categorized into low, mid and high per vessel, showed significant plaque growth for the sectors exposed to low WSS, compared to mid and high WSS (low-mid *p* = 0.025; low–high *p* = 0.024).

**Figure 4:**
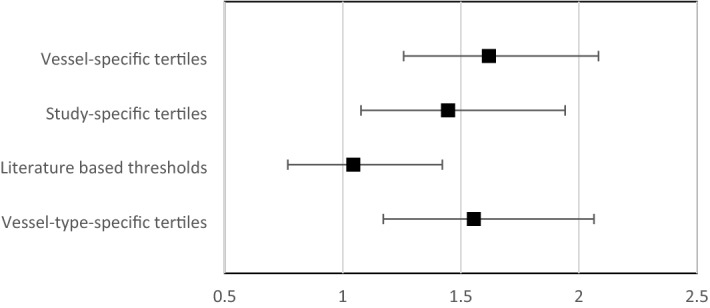
The predictive value of low WSS as predictor for plaque progression for the four different thresholding methodologies.

To gain extra insight in the predictive value of low WSS for plaque progression applying the different methodologies, the odds ratio for plaque growth of 15% PB in sectors exposed to low WSS was determined. When using the *vessel-specific* tertiles, sectors exposed to low WSS had an odds ratio (OR) of 1.6 (1.3–2.1) for plaque growth. For the *study-specific* tertiles, the OR was 1.5 (1.1–2.0), and for the *literature-based* thresholding, low WSS did not show a significant association between the exposure to low WSS and plaque growth (OR: 1.4 (1.0–2.0)) Using the *vessel-type-specific* tertiles, the OR was 1.6 (1.2–2.2) Fig. 4).

## Discussion

Wall shear stress of the blood flow at the vessel wall has been proven to impact the natural history of atherosclerotic plaque initiation, progression and destabilization. With data from recent clinical trials and current technical advances, WSS will potentially identify regions at risk of future plaque progression and cardiovascular events. However, different definitions of low and high WSS have been used across studies. Until now it is unclear how these differences impact the final outcome and should be considered when comparing the results. For the use of WSS in future clinical applications, it is essential to line up matters. Therefore, we evaluated the different definitions of low, mid and high WSS within one dataset for their impact on the estimation of plaque progression and the predictive power.

The most important findings of this study were: (1) The absolute WSS distribution majorly varies between the vessels, causing unevenly distributed low, mid and high WSS sectors between the vessels if *study-specific*, *literature-based*, *or vessel-type-specific* thresholds were used. (2) For all methodologies, sectors of low WSS showed significant plaque progression compared to mid and high WSS. However, after correcting for baseline plaque burden, only *vessel-specific* tertiles showed significantly more plaque progression at low compared to mid and high WSS. (3) The predictive power of low WSS for plaque progression compared to all the other sectors was significant using all different thresholds, except for the *literature-based* thresholds. The predictive power was most pronounced applying *vessel-specific* or *vessel-type-specific* tertiles for the definition of low WSS. (4) The effect of the definition of the WSS categories on the estimation of plaque progression varies among the different vessel types.

WSS is a continuous variable, varying in magnitude at the endothelial layer of the vessel wall with low WSS often observed close to side branches or in the inner curvature of the artery. These patched differences in wall shear stress have been related to plaque distribution and progression^14^. This study is the first showing heterogeneity in absolute WSS values among coronary arteries of different patients. Using absolute thresholds, some vessels will completely be characterized as low or high WSS. Knowing the patched pattern of atherosclerosis, these definitions might need more nuances, especially for the identification of regions that are at risk of future plaque progression. Interestingly, and similar to the earlier studies, we found independent of the definition of low WSS, low WSS always showed significant plaque progression. This explains why the different research groups, using different definitions of low WSS, still find similar results for low WSS regions being more susceptible to plaque progression^2,15,16^. Interestingly, after correction for baseline plaque burden only vessel-specific tertiles showed significant plaque progression for the sectors exposed to low WSS compared to high WSS. Furthermore, analyzing the predictive power of low WSS for plaque progression (the highest 90th percentile PB change), all but *literature-based* WSS distribution showed a significant OR, although no large differences were seen in OR between the methodologies. Whereas the absolute, *literature based*, thresholds did not show significant predictive power. The heterogeneity of the absolute WSS values might be the cause of loss in predictive power.

The absolute WSS distribution observed in the coronary arteries largely depends on the different assumptions regarding the flow and the 3D-reconstruction of the coronary arteries applied for the calculations. The used methodologies and assumptions of calculating WSS vary considerably among the different studies. Some studies use flow as derived from angiography^2^ while others use Doppler flow measurements^3,17,12^. Also, studies differ in the use of stationary flow versus pulsatile flow and multiple different flow patterns were applied as input for computational fluid dynamics. Furthermore, the 3D-reconstructions of the coronary arteries vary with respect to the presence of side branches and the used invasive imaging modalities to assess detailed information on the lumen shape (IVUS or OCT)^15,18^. If side branches are not included in the CFD model, all flow will remain in the main coronary branch and the smaller artery naturally present more distal in the vascular tree will be incorrectly classified as high WSS. Both the integration of side branches in the 3D geometry as well as patient-specific flow measurements will highly affect the calculated absolute WSS values.

In our study we used patient-tailored CFD models including side branches, with the use of patient-specific flow measurements at each single side branch. The *study-specific* thresholds that we found (low WSS: < 0.6 Pa and high WSS: > 1.14 Pa) were much lower than the literature based WSS thresholds. This might be related to the side branches that we included in our models. Interestingly, Samady et al. did include side branches in their models, however their WSS obtained in LADs were much higher than the values we found in the LAD. Speculating on the cause of this difference, one of the reasons might be the use of different inlet profiles. Samady et al. used a plug profile with velocity equal to 80% of the peak velocity measured. In this current study, a parabolic profile with a mean velocity of the peak velocity was used^7^. Both methods have been previously used, and are part of the assumptions necessary for the CFD, however could explain the overall higher WSS found in their coronary models with side branches.

If we applied the *literature-based* thresholds (low WSS: < 1 and high WSS > 2.5 Pa) to define the WSS categories, we could not demonstrate the often-observed inverse relationship between plaque progression and WSS^2,11^. So, it is advisable that if absolute thresholds are used from literature to define the WSS categories, the applied methodology for WSS calculation and the patient population is similar to the study that provides the absolute thresholds to find comparable WSS-related plaque progression.

Due to the observed considerable heterogeneity in WSS among the patients, we noticed that by applying absolute *literature-based* thresholds or *study-specific* tertiles to categorize the WSS, some vessels in this dataset qualified almost entirely as low WSS (Fig. 1). To further investigate the WSS-related heterogeneous patched distribution of plaques in those vessels, a sub-analysis of the 10 vessels with the highest number of sectors exposed to absolute low WSS (< 1 Pa, *literature-based* threshold) was performed. Interestingly, we observed that the sectors exposed to the lowest WSS in those vessels (thus *vessel-specific* low WSS) (Supplement Fig. 1), demonstrate significantly higher plaque progression than the sectors exposed to mid and high WSS. This implies that in a vessel mainly exposed to absolute low WSS, there is still a significant variation in plaque progression between the sectors exposed to relatively low and high WSS within the vessel.

It is known that the three main coronary arteries have geometrical and hemodynamical differences^19^. Therefore, we hypothesized that the definition of low and high WSS to predict plaque progression and destabilization could be different among the different vessel types. Indeed, both the WSS-related plaque progression and the predictive power of low WSS for plaque progression differed considerably among the different vessel types. The absolute WSS values differed significantly between the three vessels, with the LAD having the lowest absolute WSS values and the LCX the highest absolute shear stress values. These differences are of substantial influence when dividing the sectors according to *study-specific* thresholds or *literature-based* thresholds, especially for the association of low WSS with plaque progression. Although the LCX showed the highest absolute WSS, sectors showed more plaque progression than the LAD, being exposed to lower absolute WSS. The variations in absolute plaque growth and WSS-related plaque growth in these different vessel types might be attributed to differences in local hemodynamics^19–21^, geometrical differences concerning curvature and number of side branches^22^. Unfortunately, the number of coronary arteries investigated in this study was too small to allow a detailed analysis of these differences.

As described above, the absolute WSS is affected by the flow and therefore patient-specific flow measurements are often used in the CFD simulations to obtain accurate absolute WSS values. However, flow measurements are labor-intensive and challenging in clinical practice. With the use of *vessel-specific* thresholds, the lowest one-third WSS at the vessel wall will always be allocated as low WSS, regardless of the magnitude of the WSS, with the benefit that invasive flow measurements become redundant. The absolute measured velocity might become subordinate to the lumen geometry of the reconstructed vessel, for the allocation of low and high WSS. This is attractive in case the flow measurements are not available, for instance, in retrospective studies. Since this study showed that WSS-related plaque progression as based on vessel-specific WSS thresholds was not inferior to *study-specific* WSS thresholds, future research might adopt this approach to identify regions at risk. However, when using assumptions for the flow, the absolute WSS values among the patients cannot be compared which in future studies with other objectives could be required.

## Conclusion

In this study different methodologies were applied to define low, mid and high WSS in human coronary arteries of acute coronary syndrome patients. We studied the predictive power of low WSS using the different definitions of low WSS for plaque progression within one patient group. On population level, the established patterns of most plaque progression at regions exposed to low WSS are visible using all the four different methodologies to define low WSS. However, on an individual basis with measured vessel-specific flow, WSS values vary too widely to use absolute thresholds for determining low WSS when used to identify regions at risk of plaque progression. Depending on the research question, using a relative measure by dividing coronary arteries in *vessel-specific* tertiles is a robust methodology to predict local plaque progression.

## Methods

### Patient population

This single-center study was designed to evaluate the association between biomechanical parameters and natural history of atherosclerotic disease in non-stented coronary arteries. Patients presented with an acute coronary syndrome (ACS), with at least one non-stented non-culprit coronary vessel, were eligible for enrollment. The most important clinical exclusion criteria were: previous coronary artery bypass graft surgery, 3-vessel disease, atrial fibrillation, left ventricular ejection fraction < 30%, and renal insufficiency (creatinine clearing < 50 ml/min). Written informed consent was obtained from all patients. The local medical ethical committee of the Erasmus MC (MED 2015-535, NL54519.078.15) approved the study protocol and was registered (ISCRTN:43170100). The study was conducted in accordance with the World Medical Association Declaration of Helsinki (64th WMA General Assembly, Fortaleza, Brazil, October 2013) and Medical Research Involving Human Subject Act (WMO).

### Clinical data acquisition

After successful percutaneous coronary intervention, a study segment with a length of at least 30 mm and two visible side branches (diameter > 1.5 mm) was selected. Intravascular imaging of the study segment was performed using intravascular-ultrasound (IVUS) (TVC Insight Coronary Imaging Catheter, InfraRedX, Burlington, MA, USA) (0.5 mm/sec) at baseline and repeated at 12 months follow-up. Furthermore, invasive local flow measurements at multiple locations between the side branches in the study segment were preformed to assess patient-specific blood flow at each location of interest using a ComboWire (Phillips Volcano, Zaventem, Belgium). One month after the index procedure, an ECG-triggered coronary computed tomography angiography (CCTA) was performed. (SOMATOM Force (192 slice 3rd generation dual-source CT scanner), Siemens Healthineers, Germany).

### Image analysis and the 3D reconstructions

**Figure 5:**
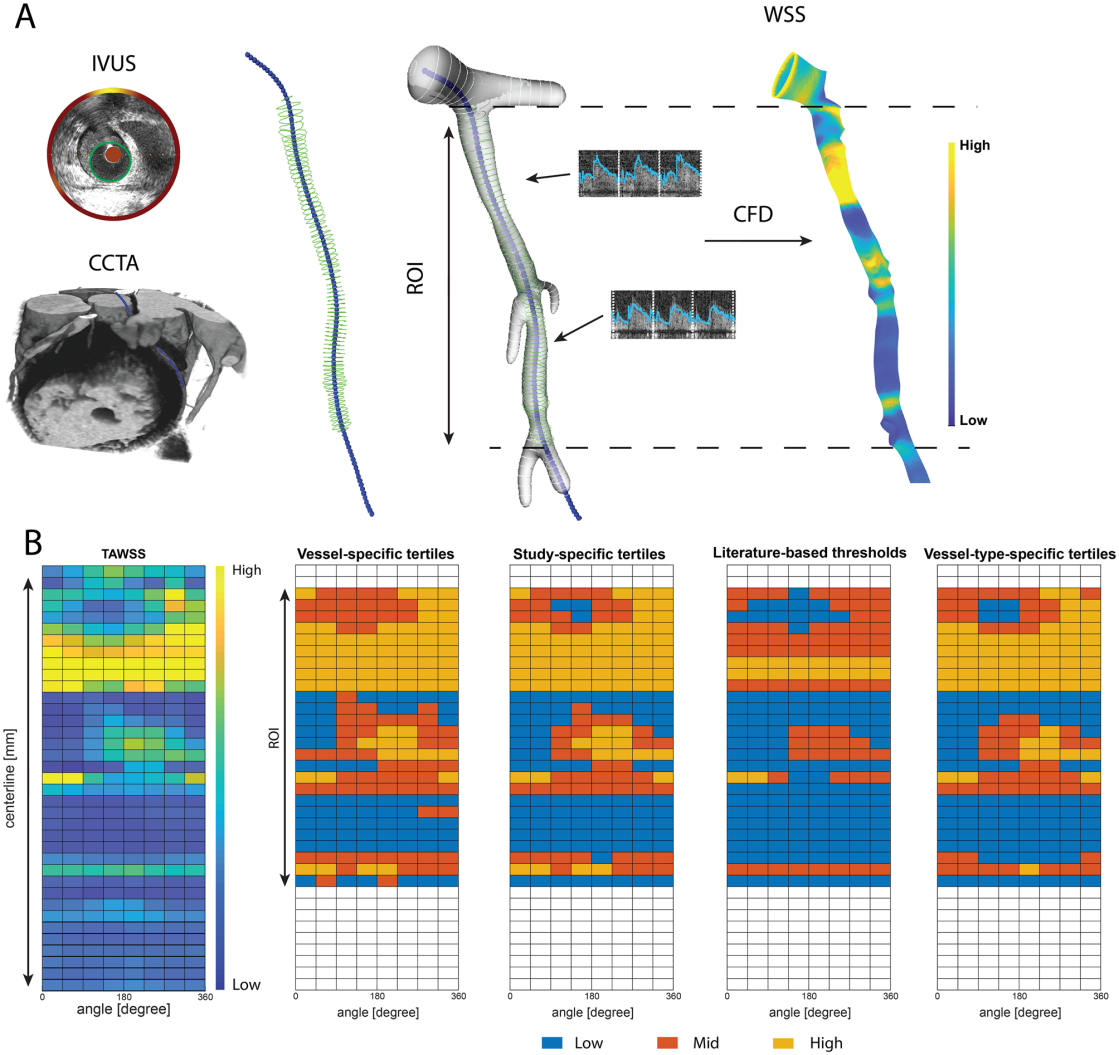
Example of an LCX showing the methodology of 3D-reconstruction of coronary and wall shear stress (WSS) calculations. (**A**) Lumen (green) was delineated in intravascular ultrasound (IVUS) images and merged with the centerline (blue markers) segmented from the coronary computer tomography angiography (CCTA) to create a 3D reconstruction. WSS calculations were performed using local flow measurements and computational fluid dynamics, resulting in local WSS. (**B**) The 3D reconstruction was converted in a 2D-map by folding the vessel open in the longitudinal direction. Creating a 2D map, dividing the vessel into sectors of 45°/1.5 mm thick*,* WSS was averaged for each sector*.* For each methodology (vessel-specific tertiles, study-specific tertiles, literature-based thresholds (low WSS < 1.0 Pa) and vessel-type-specific tertiles) a 2D map was created and depicting the distribution of WSS in low (blue), mid (orange) and high (yellow) WSS. Images were generated by MevisLab (MeVis Medical Solutions AG, Bremen, Germany) and MATLAB (v 2017B, Mathworks Inc, USA).

The methodology used to create the three-dimensional (3D) vessel reconstructions, as well as the computational fluid dynamic (CFD) analysis were previously described in great detail^4,23^. In brief, by fusing the 3D spatial information of the coronary vessel centerline segmented from the CCTA and the lumen contours extracted from the IVUS, a 3D-reconstruction was made in MeVisLab (MeVis Medical Solutions AG, Bremen, Germany) (Fig. 5). The CCTA scan was reconstructed at diastole to ensure maximal filling of the coronary arteries, including all the side branches.

In the IVUS analysis, to remove diameter variations as observed in the images obtained by a continuous IVUS pullback due to cardiac contraction, the images were gated by using an in-house developed MATLAB (v 2017B, Mathworks Inc, USA) algorithm. The gated frames corresponded with the end-diastolic phase of the cardiac cycle. To evaluate plaque progression, both lumen and the external elastic membrane (EEM) contours were segmented in the gated IVUS frames of the study segment. IVUS lumen was used for the 3D-reconstruction, where lumen and EEM were used to evaluate plaque size and plaque progression. An intra-observer analysis was performed in a random sample of 5 IVUS pullbacks (748 frames) with at least 2 months interval between the segmentations. A good reproducibility of EEM area, lumen area and plaque area was found (0.996 [95%CI 0.996–0.997], 0.983[95%CI 0.963–0.990] and 0.958 [95%CI 0.939–0.970]). In IVUS images, calcium is defined as a bright signal with a dark shadow behind it. Calcium was identified as angles in the frames, and regions with extensive calcifications (> 90 degrees) were excluded from plaque progression analysis.

Matching of IVUS contours and CCTA was done using side branches visible in both modalities, and corrected for longitudinal and circumferential orientation as previously described and validated by van der Giessen et al.^24^. The IVUS lumen was placed perpendicular to the centreline. For subsequent computational fluid dynamics (CFD), reliable inlet and outlets were needed. Therefore, to create a model that reassembles real-life anatomy in detail, the side branches (> 1.5 mm) as well as the regions proximal and distal of the IVUS-derived region of interest (ROI) were segmented in the CCTA, scaled to the IVUS lumen contours and fused with the 3D-reconstruction. To finalize the model, a surface was generated by interpolating the contours of the 3D-reconstruction. In each 3D-reconstructed geometry, a time-dependent CFD simulation was performed, assuming blood as an incompressible fluid (Fluent, v.17.1, ANSYS Inc.). Each 3D-reconstruction was converted to a tetrahedral mesh in ICEM CFD (V.17.1 ANSYS INC) with a 5-layer prism layer at the boundary to accurately assess the wall shear stress. The mesh size was determined by a mesh independence study (only errors within 1% of shear stress were allowed), which resulted in a minimum element size of 0.064 mm and a maximum element size of 0.16 mm. Additionally, blood was assumed to behave as a shear-thinning, non-Newtonian fluid and the Carreau model ($\mu_{0}$: 0.25 Pa·s, $\mu_{\infty }$: 0.0035 Pa·s n: 0.25 and $\lambda$: 25 s) was used in the simulations^25,26^. The most proximal invasive flow measurement of good quality was used to prescribe time-varying inlet boundary condition in the CFD simulation. Furthermore, for the outlet boundary conditions, the flow distribution through the side branches was calculated based on the flow measurements at different locations in the coronary artery. For the regions with no reliable flow measures, a previously described scaling law was used to determine the flow ratio between the mother and her side branches. Additionally, the vessel lumen was considered as rigid and subjected to no-slip condition. Each To obtain the time-averaged WSS, the computed shear stresses were averaged over a cardiac cycle.

### Data analysis

All IVUS-derived data, such as calcification, wall thickness based on the EEM (baseline and follow-up), were mapped on the 3D geometry of the coronary vessel using VMTK (Orobix, Bergamo, Italy) and MATLAB (v2017b, Matworks Inc, Natick, MA, USA). To perform statistical analysis, the 3D reconstruction was converted into a 2D-map by folding the vessel open in the longitudinal direction. In this 2D map configuration, the arteries were divided into cross-sectional segments of 1.5 mm thick. Subsequently, these segments were divided into 8 sectors of 45° with the lumen center as intersection. The WSS was averaged for each sector and categorized by using the three different methodologies to define low, mid and high WSS. First, for *vessel-specific* thresholds, the sectors of one vessel were equally divided into three groups based on the WSS, creating *vessel-specific* tertiles. Second, for the *study-specific* thresholds, the sectors of all vessels combined were equally divided into three groups, allocating low, mid and high WSS to one third of all the sectors. Third, all sectors of all vessels were categorized using absolute WSS thresholds based on literature-based values. All sectors below 1 Pa were categorized as low WSS, between 1 and 2.5 Pa as mid WSS and above 2.5 Pa as high WSS^3^. Finally, *vessel-type-specific* thresholds were determined. Therefore all sectors of all LAD vessels were equally divided into three groups, allocating low, mid, and high WSS to one-third of the sectors. Similarly, thresholds were extracted for the LCX and the RCA. Furthermore, analysis on plaque progression was done by mapping the IVUS-based vessel area (based on the EEM) and lumen area and the derived wall thickness on these 2D-maps. Both baseline and follow-up plaque burden (PB) were calculated by sectorial plaque area/sectorial vessel area*100% and subtracted to obtain the plaque progression over time.

### Statistics

IBM SPSS statistics (version 25.0) software was used for the statistical analysis. Normally distributed data was presented as mean ± standard deviation, statistical differences was determined with a student t-test or ANOVA with post hoc testing (Bonferroni). Non-normally distributed data were displayed as median (interquartile range) and significance was tested using a Mann–Whitney U test. All statistical analysis of change in PB (follow-up-baseline) was done with a mixed model. The WSS category (low, mid and high) and vessel type (LAD, LCX or RCA) as a fixed factor and the individual vessel as a random factor to take the within-subject-correlation into account. Furthermore, per vessel, a random sample of 25% of the sectors was used in the mixed model to minimize possible dependencies in the dataset. The mixed model analyses were performed with and without correction for baseline plaque burden. The results showing the relationship between WSS and plaque progression are presented as estimated means of the PB change and the 95% confidence interval (95%CI) and significance between the groups was determined after post hoc testing (Bonferroni). To investigate the predictive value of low WSS, a generalized linear mixed-effect model was used, with the individual vessel as a random factor. Plaque growth was defined as delta PB of 15% and is based on the 90^th^ percentile of delta PB of all available sectors of all patients. *P* < 0.05 was considered significant in all tests.

## Supplementary Information

Supplementary Legends.

Supplementary Figure 1.

Supplementary Figure 2.

Supplementary Figure 3.

Supplementary Tables.
