# Supplementary material for: The definition of low wall shear stress and its effect on plaque progression estimation in human coronary arteries
Source: Sci Rep. 2021 Nov 11;11:22086. doi: 10.1038/s41598-021-01232-3 (PMC8586146; doi:10.1038/s41598-021-01232-3)
Supplement: Supplementary file 1 — Supplementary Legends. [file 41598_2021_1232_MOESM1_ESM.docx]

**Supplement figure 1.**

Plaque progression of sectors by using the relative vessel-specific tertiles in the 10 vessels with the lowest absolute wall shear stress.

**Supplement figure 2**

Wall shear stress distribution and plaque progression for the four different thresholding methodologies, corrected for baseline plaque burden. * p<0.05

**Supplement figure 3**

Wall shear stress distribution and plaque progression for the four different thresholding methodologies split up in the three coronary artery types, corrected for baseline plaque burden. * p<0.05
