# Supplementary figures and images for: The definition of low wall shear stress and its effect on plaque progression estimation in human coronary arteries
Source: Sci Rep. 2021 Nov 11;11:22086. doi: 10.1038/s41598-021-01232-3 (PMC8586146; doi:10.1038/s41598-021-01232-3)

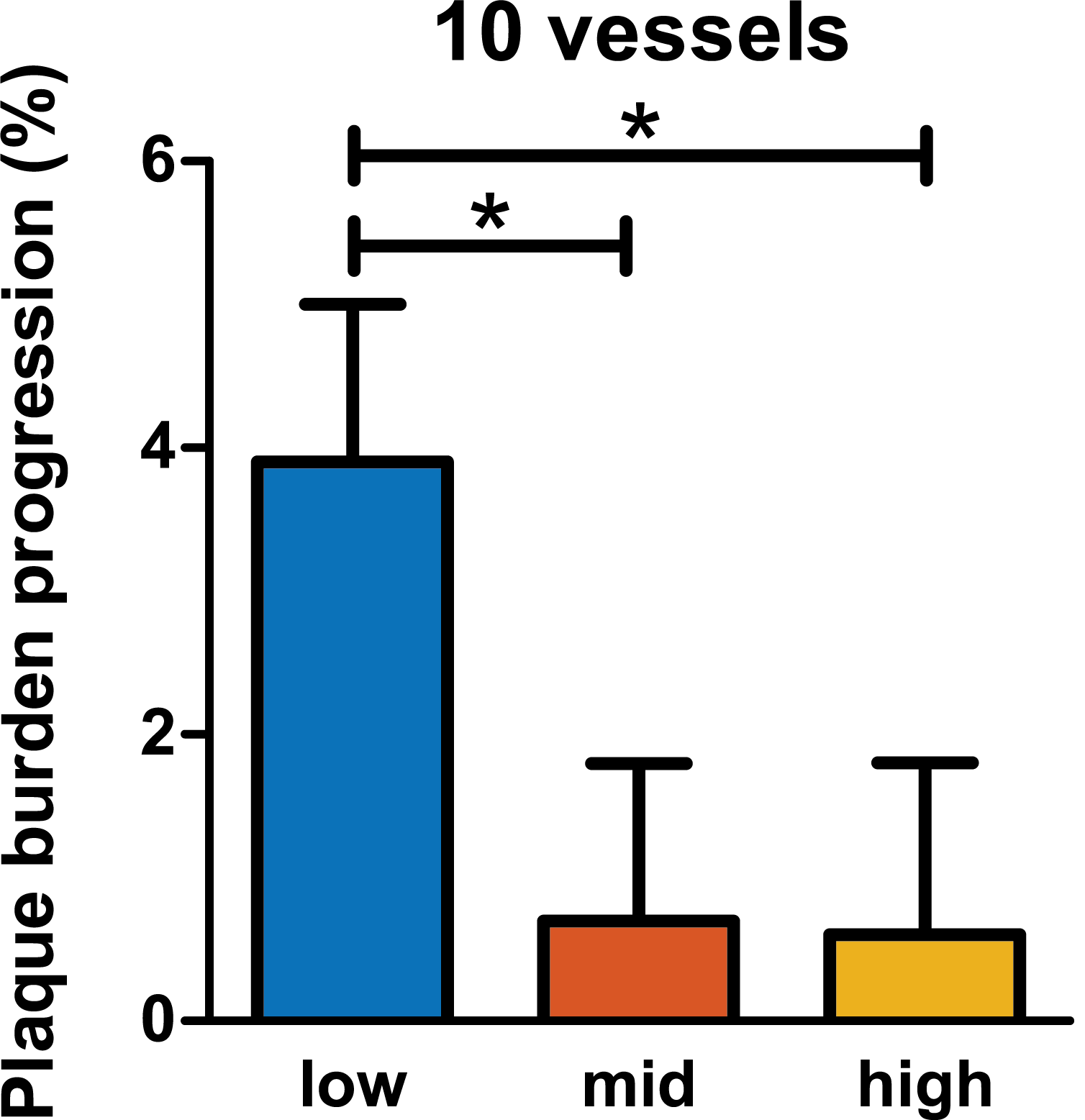

Supplement: Supplementary file 2 — Supplementary Figure 1. [file 41598_2021_1232_MOESM2_ESM.png]

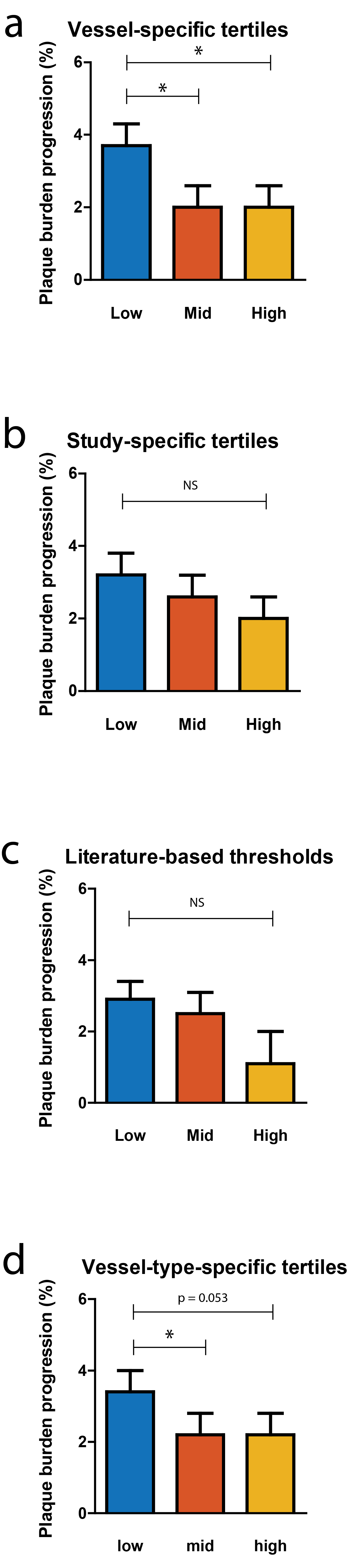

Supplement: Supplementary file 3 — Supplementary Figure 2. [file 41598_2021_1232_MOESM3_ESM.png]

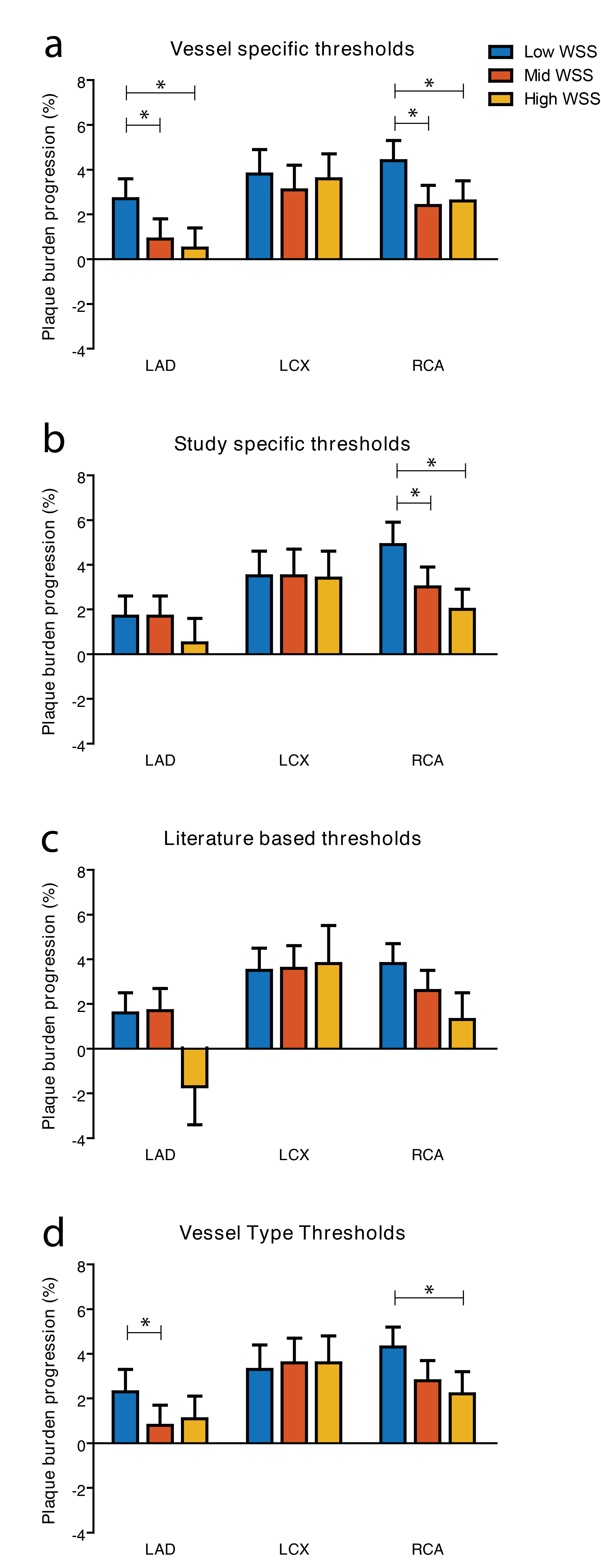

Supplement: Supplementary file 4 — Supplementary Figure 3. [file 41598_2021_1232_MOESM4_ESM.png]
