# Supplementary material for: The definition of low wall shear stress and its effect on plaque progression estimation in human coronary arteries
Source: Sci Rep. 2021 Nov 11;11:22086. doi: 10.1038/s41598-021-01232-3 (PMC8586146; doi:10.1038/s41598-021-01232-3)
Supplement: Supplementary file 5 — Supplementary Tables. [file 41598_2021_1232_MOESM5_ESM.docx]

| **Table Supplement. Vessel-specific thresholds** | | | |
| --- | --- | --- | --- |
|  | | | |
| Vessel | Low-Mid threshold (Pa) | Mid-High threshold(Pa) | VesselType |
| 1 | 0.25 | 0.36 | LAD |
| 2 | 0.27 | 0.36 | LAD |
| 3 | 0.34 | 0.52 | LAD |
| 4 | 0.34 | 0.52 | RCA |
| 5 | 0.35 | 0.46 | LAD |
| 6 | 0.37 | 0.51 | LAD |
| 7 | 0.38 | 0.57 | RCA |
| 8 | 0.40 | 0.62 | LCX |
| 9 | 0.42 | 1.34 | LCX |
| 10 | 0.43 | 1.21 | LCX |
| 11 | 0.51 | 0.75 | LAD |
| 12 | 0.51 | 0.66 | RCA |
| 13 | 0.53 | 0.87 | LAD |
| 14 | 0.54 | 0.84 | LAD |
| 15 | 0.55 | 2.05 | LCX |
| 16 | 0.56 | 0.80 | RCA |
| 17 | 0.60 | 0.96 | LCX |
| 18 | 0.60 | 1.17 | RCA |
| 19 | 0.61 | 1.22 | RCA |
| 20 | 0.61 | 0.82 | RCA |
| 21 | 0.63 | 0.78 | LAD |
| 22 | 0.64 | 0.94 | LCX |
| 23 | 0.65 | 0.93 | LAD |
| 24 | 0.66 | 1.73 | RCA |
| 25 | 0.70 | 1.20 | LCX |
| 26 | 0.71 | 0.92 | RCA |
| 27 | 0.76 | 1.10 | RCA |
| 28 | 0.78 | 1.45 | LCX |
| 29 | 0.79 | 1.13 | RCA |
| 30 | 0.96 | 1.56 | LAD |
| 31 | 0.98 | 1.52 | LAD |
| 32 | 1.00 | 1.43 | RCA |
| 33 | 1.03 | 1.36 | LCX |
| 34 | 1.15 | 1.85 | RCA |
| 35 | 1.20 | 2.21 | RCA |
| 36 | 1.27 | 1.98 | LCX |
| 37 | 1.28 | 2.39 | LAD |
| 38 | 1.34 | 2.51 | LCX |
| 39 | 1.42 | 2.34 | RCA |
| 40 | 1.61 | 2.26 | RCA |
| 41 | 1.99 | 3.69 | LAD |
| LAD = left anterior descending, LCX = left coronary circumflex, RCA = right coronary artery | | | |
